# Cervical cancer risk factors among HIV-infected Nigerian women

**DOI:** 10.1186/1471-2458-13-582

**Published:** 2013-06-14

**Authors:** Uzoma Ononogbu, Maryam Almujtaba, Fatima Modibbo, Ishak Lawal, Richard Offiong, Olayinka Olaniyan, Patrick Dakum, Donna Spiegelman, William Blattner, Clement Adebamowo

**Affiliations:** 1 Department of Epidemiology, Harvard School of Public Health, Boston, MA 02115, USA; 2 Office of Research and Training, Institute of Human Virology, Abuja, Nigeria; 3 National Hospital, Abuja, Nigeria; 4 University of Abuja Teaching Hospital, Gwagwalada, Nigeria; 5 Department of Biostatistics, Harvard School of Public Health, Boston, MA 02115, USA; 6 Department of Epidemiology and Public Health, Institute of Human Virology and Greenebaum Cancer Centre, University of Maryland School of Medicine, Baltimore, MD 21201, USA

**Keywords:** Cervical cancer, Screen and treat, HIV, VIA/VILI

## Abstract

**Background:**

Cervical cancer is the third most common cancer among women worldwide, and in Nigeria it is the second most common female cancer. Cervical cancer is an AIDS-defining cancer; however, HIV only marginally increases the risk of cervical pre-cancer and cancer. In this study, we examine the risk factors for cervical pre-cancer and cancer among HIV-positive women screened for cervical cancer at two medical institutions in Abuja, Nigeria.

**Methods:**

A total of 2,501 HIV-positive women participating in the cervical cancer screen-and-treat program in Abuja, Nigeria consented to this study and provided socio-demographic and clinical information. Log-binomial models were used to calculate relative risk (RR) and 95% confidence intervals (95%CI) for the risk factors of cervical pre-cancer and cancer.

**Results:**

There was a 6% prevalence of cervical pre-cancer and cancer in the study population of HIV-positive women. The risk of screening positivity or invasive cancer diagnosis reduced with increasing age, with women aged 40 years and older having the lowest risk (RR=0.4; 95%CI=0.2–0.7). Women with a CD4 count of 650 per mm^3^ or more also had lower risk of screening positivity or invasive cancer diagnosis (RR=0.3, 95%CI=0.2–0.6). Other factors such as having had 5 or more abortions (RR=1.8, 95%CI=1.0–3.6) and the presence of other vaginal wall abnormalities (RR=1.9, 95%CI=1.3–2.8) were associated with screening positivity or invasive cancer diagnosis.

**Conclusion:**

The prevalence of screening positive lesions or cervical cancer was lower than most previous reports from Africa. HIV-positive Nigerian women were at a marginally increased risk of cervical pre-cancer and cancer. These findings highlight the need for more epidemiological studies of cervical cancer and pre-cancerous lesions among HIV-positive women in Africa and an improved understanding of incidence and risk factors.

## Background

In 2008, cervical cancer was the third most common cancer among women and the seventh most common cancer overall with 530,000 new cases and 275,000 deaths reported [1]. Women in Sub-Saharan Africa are disproportionately affected, where it is the most common cancer in women, accounting for 13% of all female cancers [1,2]. In Nigeria, it is the second most common female cancer after breast cancer, with an age standardized incidence rate of 34.5 cases per 100,000 women in 2010 [3].

Virtually all cases of cervical pre-cancer and cancer are associated with a high-risk human papillomavirus (hrHPV) infection, with types 16 and 18 reported to account for the majority of cases [4,5]. However, only about 12% of individuals with persistent hrHPV infection go on to develop cervical pre-cancer and cancer. Hence, hrHPV infection can a cause of cervical cancer, but is not the exclusive cause. In addition to hrHPV, other factors impact progression, from persistent hrHPV infection to cervical pre-cancer to cancer. These include smoking, parity, education, diet, physical inactivity, sexual behavior and use of oral contraceptives [6,7]. Other factors, including population growth and aging, are also contributing to the rising burden of cervical cancer in developing countries [8].

Since the onset of the HIV epidemic, the United States Centers for Disease Control and Prevention (CDC) has classified cervical cancer, Kaposi sarcoma and non-Hodgkin’s lymphoma as AIDS-defining cancers because of their close association with HIV infection [2]. However, unlike the latter two types of cancer, the risk of cervical cancer is only marginally elevated at best among HIV-infected women [9-13]. Furthermore, compared with other AIDS-defining cancers, the incidence of cervical cancer has not decreased substantially with increasing use of anti-retroviral therapy, which suggests that the role of immunosuppression in the progression to invasive cancer may be marginal [9,14-18].

In developed countries, some 75% of women are screened—typically by Papanicolaou staining (Pap smears) and more recently by human papillomavirus (HPV) DNA-based tests—compared with 5% in developing countries [19]. As a consequence, the incidence of cervical cancer in developed countries has been reduced substantially, in contrast to developing countries such as Nigeria, where the incidence has remained stable [3,20]. The choice of screening method for cervical cancer is also a challenge for developing countries. Widespread adoption of Pap smears has not been feasible in developing countries because of the need for high quality cytology equipment, laboratories and well-trained clinicians who can interpret Pap smear results, the need for multiple hospital visits, and the need for follow-ups and the recall of patients several times [19,21]. HPV DNA testing is expensive, requires multiple visits and is beyond the resources of most low- and middle-income countries (LMIC) at the present time. Other factors, including poor health care infrastructure, lack of resources and competing needs for limited health care resources, also mitigate against widespread adoption of cervical cancer screening in low resource countries [19].

An alternative cervical cancer screening method that is recommended for use in developing countries is visual inspection with acetic acid or Lugol’s iodine (VIA/VILI). VIA/VILI is an inexpensive test that can be easily adopted and scaled up in the healthcare system of LMIC, does not require specialized laboratories or equipment, can be performed by a wide range of health care professionals after a short period of training and does not require several visits by patients [10,16,19,22]. VIA/VILI is increasingly used as a “screen and treat” approach to cervical cancer screening in LMIC where treatment for abnormal lesions is performed during one single clinic visit, thereby minimizing the need of patients having to attend a follow-up [16,19].

In some countries, donor funding from the President’s Emergency Plan for AIDS Relief (PEPFAR) and the Global Fund has provided the resources and infrastructure needed to scale up visual inspection with acetic acid testing (VIA) [16]. For example, Zambia leveraged these resources, rapidly expanded its VIA model and integrated it into the health care system. This has ensured the success of the program without placing extra burden on the health care system, thus maximizing the potential of the “screen and treat” approach [10,16,22]. VIA is reported to have 80% sensitivity, 92% specificity, a 10% positive predictive value and a 99% negative predictive value [21]. In this paper, we report risk factors for VIA/VILI positivity among the study’s screened population in Nigeria.

## Methods

### Place of study

This study was carried out at the University of Abuja Teaching Hospital and the National Hospital, both in Abuja, Nigeria.

### Screening population

HIV-positive women older than 18 years of age either on anti-retroviral therapy (ART) or ART naïve attending PEPFAR treatment clinics for HIV care were enrolled at both sites. Participants were women who voluntarily presented and gave informed consent for cervical cancer screening.

### Screening procedure

After obtaining informed consent, nurses obtained demographic and clinical information on all participants and examined the introitus and vulva, noting any abnormalities. A bivalve speculum was then introduced into the vagina for examination of the cervix using a halogen lamp. The squamo-columnar junction of the cervix (transformation zone) was identified and any secretions or exudate cleaned off before a nurse applied a cotton wool swab soaked in 5% acetic acid solution for 3 minutes. The results of each examination were noted. Pre-cancerous lesions were defined as being dense aceto-white lesions with well-defined margins observed within the vicinity of the transformation zone originating from the squamo-columnar junction, or if the whole cervix or cervical growth turned white. A suspicion of cancer was defined as any cervical ulcer or growth being observed [23]. Following VIA, if there was any uncertainty about the lesion observed, visual inspection with Lugol’s iodine (VILI) was conducted. A positive VILI was characterized as being well-defined, bright yellow iodine non-uptake areas touching the squamo-columnar junction or close to the cervical os if the squamo-columnar junction was not seen.

Results of VIA or VILI were classified according to the International Agency for Research Against Cancer (IARC) manual and recorded after each test [24]. Positive areas were mapped on case report forms and the number of positive lesions, the quadrant affected and degree of extension noted. Digital images (cervigrams) of the cervix were captured for all screened women regardless of the outcome of VIA using off-the-shelf digital cameras (Canon Powershot SX20 IS®). Cervigrams were used to provide patients with immediate feedback on the results of their examination, and were saved on a computer as part of the client’s record. They were subsequently reviewed by a consulting gynecologist for quality assurance purposes.

Women with confirmed VIA or VILI positive results were offered immediate treatment with cold coagulation. This is an ablative therapy used for treatment of intraepithelial neoplasia that is not suspected of being cancerous, not covering more than 75% of the cervix, is not disappearing into the endo-cervical canal, and the squamo-columnar junction is fully visible. A patient with a cervix suspicious for cancer or with intraepithelial neoplasia was ineligible for cold coagulation according to predefined criteria. These women were referred to the gynecology clinic to undergo colposcopy and biopsy to confirm probable cancer diagnosis and to receive further treatment, as is standard practice in screen and treat programs [23].

### Quality assurance

At the end of every week, quality assurance meetings were held with nurses and the consulting gynecologist to review all cervigrams taken during the week, with the aim of assessing the nurses’ visual detection skills and referral decisions. In those cases where there was a missed diagnosis of clinical relevance the patient was recalled.

### Statistical analysis

Relative risk and 95% confidence intervals (95%CI) were calculated using log-binomial regression models [25,26]. The p-value for trend for ordinal variables was calculated using the median value of each category. A liberal criterion for the selection of potential confounders and possible independent risk factors was employed; all variables that were significant in the univariate models at p≤0.20 were included in the multivariate models [27]. We divided the continuous variables (age and last CD4 count) by multiples of five, which corresponded approximately to quartiles of the age distribution. We investigated possible non-linearity in the log-binomial models between screening status and continuous variables (age, age at first sexual intercourse, total number of pregnancies, total number of abortions and last CD4 count) using stepwise restricted cubic splines. A likelihood ratio test was used to compare the log-linear model to the model with any non-linear terms that were selected in the smoothing process [28,29]. All data analysis was performed using SAS 9.3 (SAS Institute; Cary, NC, USA).

## Results

Socio-demographic and clinical characteristics of the study participants are shown in Tables 1 and 2. A total of 2,501 HIV-infected women on ART were screened between 2010 and 2012. The mean age and standard deviation (SD; the second number) was 35 (7) years. The mean number of sexual partners (SD) was 1 (1). The mean age (SD) at first sexual intercourse was 19 (4) years. The mean (SD) number of pregnancies was 4 (2). The mean (SD) number of abortions experienced was 2 (2). Christianity was the most common religion, with 84% of the women identifying as Christians. The level of education within this group of women was high, with 77% having secondary education or higher. The majority of women, 52%, were married at the time of the study. Most women, 77%, had CD4 counts above 300; the median CD4 count was 451 cells per mm^3^. The majority of the HIV-infected women, 86%, were asymptomatic and were classified as having Stage 1 at baseline as defined by World Health Organization guidelines [30].

**Table 1:** Socio-demographic characteristics of HIV-infected women (n=2,501)

	**n (%)**	**Missing (%)**
Age category (years)		11 (0.4)
<30	504 (20)	
30–34	696 (28)	
35–39	663 (27)	
≥40	627 (25)	
Age at first sexual intercourse (years)		29 (1.2)
<15	156 (6)	
15–19	1242 (50)	
20–24	856 (35)	
≥25	218 (9)	
Number of sexual partners		10 (0.4)
≤2	2400 (96)	
3–4	68 (3)	
≥5	23 (1)	
Total number of pregnancies^1^		1 (0.04)
0–2	722 (29)	
3–4	827 (33)	
5–6	571 (23)	
≥7	380 (15)	
Total number of abortions		6 (0.2)
0–2	1873 (75)	
3–4	445 (18)	
≥5	177 (7)	
Religion		5 (0.2)
Islam	404 (16)	
Christian	2092 (84)	
Education		2 (0.1)
None	150 (6)	
Quaranic	27 (1)	
Primary	402 (16)	
Secondary	773 (31)	
Tertiary	1147 (46)	
Marital Status		0 (0)
Single	443 (18)	
Married	1297 (52)	
Widowed	537 (21)	
Divorced	207 (8)	
Cohabiting	17 (1)	
Contraceptives use^2^		3 (0.1)
Yes	657 (26)	
No	1841 (74)	
Hormonal contraceptives^3^		0 (0)
Yes	29 (1)	
No	2472 (99)	
Intrauterine devices		1 (0.04)
Yes	32 (1)	
No		2468 (99)
Barrier contraceptives		0 (0)
Yes	554 (22)	
No	1947 (78)	
Tubal ligation		1 (0.04)
Yes	11 (0)	
No	2489 (100)	

^1^Total number of pregnancies is defined as pregnancies carried after 28-weeks, regardless of outcome.

^2^Contraceptive use refers to history of contraceptive use.

^3^Hormonal contraceptives includes oral contraceptives, hormonal injections and intradermal implants.

**Table 2:** Clinical characteristics of HIV-infected women (n=2,501)

	**n (%)**	**Missing (%)**
BMI (kg/m^2^)		1840 (73.6)
<18.5	46 (7)	
18.5–24.9	401 (61)	
25–29.9	49 (7)	
≥30	165 (25)	
Vaginal wall abnormality		0 (0)
None	2241 (90)	
Other^1^	260 (10)	
VIA		0 (0)
Negative	2344 (94)	
Positive	155 (6)	
Invasive cancer	2 (0)	
Last CD4 count (per mm^3^)		365 (14.6)
<300	490 (23)	
300–<450	560 (26)	
450–<650	592 (28)	
≥650	494 (23)	
WHO stage		1118 (44.7)
Stage 1	1185 (86)	
Stage 2	104 (7)	
Stage 3	84 (6)	
Stage 4	10 (1)	
ART regimen currently on^2^		1008 (40)
TDF-containing ART^3^	778 (52)	
ZDV-containing ART^4^	600 (40)	
d4T-containing ART^5^	115 (8)	

^1^Other includes discharge, lesion, warts, lesion and warts, warts and discharge, and other observations.

^2^All women were on ART, the regimen information here is based on those that were verified from clinical records.

^3^TDF: Tenofovir.

^4^ZDV: Zidovudine.

^5^d4T: Stavudine.

**Figure 1:**
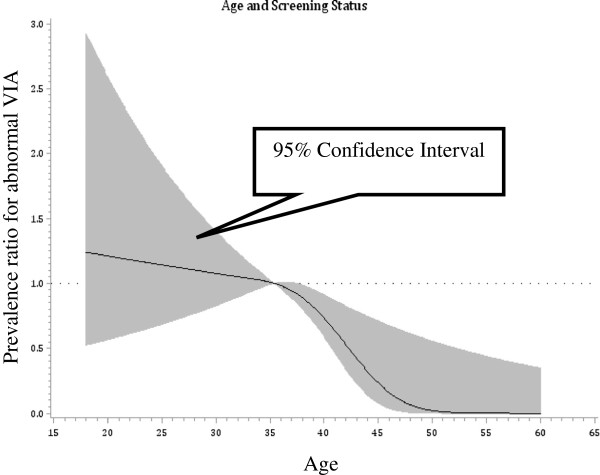
Relationship between age and screening status of HIV-infected women participating in cervical cancer screening in Abuja, Nigeria 2012.

Few participants, 6%, had a positive screening test result or a confirmed diagnosis of invasive cervical cancer. Women with positive results and invasive cancer were grouped together for analysis; although we recognize that a positive VIA/VILI result does not necessarily equate to a cervical cancer diagnosis. The results of the univariate associations are shown in Table 3. The median age of women with a positive screening result/invasive cancer was 32 years compared with a median of 35 years in women with a negative screening result (p_trend_ < 0.0001). The median number of pregnancies among women with a positive VIA result/invasive cancer was 3 compared with 4 pregnancies in women with a negative (p_trend_=0.03). A greater proportion of women with VIA positive lesions were Christians (p=0.04). Women with a positive screening result/invasive cancer also had a higher proportion of vaginal wall abnormality (p=0.0006). The most recent CD4 count prior to screening was significantly higher among women with a negative screening result compared with women with a positive screening result/invasive cancer (p <0.0001). The results of the multivariate analyses are shown in Table 3. The risk of screening positivity or invasive cancer diagnosis decreased with increasing age, with women aged 40 years and older having the lowest risk (RR=0.4; 95%CI=0.2–0.7). Women with a CD4 count of 650 per mm^3^ or more had the lowest risk of screening positivity or invasive cancer diagnosis (RR=0.3, 95%CI=0.2–0.6). Other independent risk factors, such as having had five or more abortions (RR=1.8, 95%CI=1.0–3.6) and the presence of other vaginal wall abnormalities (RR=1.9, 95%CI=1.3–2.8), were associated with screening positivity or invasive cancer diagnosis. There were no independent associations with age at first sexual intercourse or multiple pregnancies. We detected non-linearity in the relationship between age and screening status, where risk was slightly elevated up to age 35 and then declined dramatically up to age 50, after which no further decrease in risk was apparent (Figure 1).

**Table 3:** Association between socio-demographic and clinical characteristics and a positive cervical screening test among HIV-infected women

	**Univariate**		**Multivariate**	
	**RR (95%CI)**	**p-value**	**RR (95%CI)**	**p-value**
**Socio-demographic characteristics**				
Age category (years)		<0.0001*		0.0005*
<30 (ref)	1.0		1.0	
30–34	0.8 (0.5–1.1)		(0.6–1.3)	
35–39	0.6 (0.4–0.9)		(0.5–1.1)	
≥40	0.3 (0.2–0.5)		0.4 (0.2–0.7)	
Age at first sexual intercourse (years)		0.06*		0.05*
<15 (ref)	1.0		1.0	
15–19	0.9 (0.5–1.6)		0.8 (0.5–1.3)	
20–24	0.7 (0.4–1.3)		0.6 (0.4–1.1)	
≥25	0.6 (0.2–1.3)		0.5 (0.2–1.2)	
Number of sexual partners		0.2*		
≤2 (ref)	1.0			
3–4	1.2 (0.5–2.8)			
≥5	2.1 (0.7–6.1)			
Total number of pregnancies		0.03*		0.1*
0–2 (ref)	1.0		1.0	
3–4	0.8 (0.5–1.1)		0.7 (0.5–1.1)	
5–6	0.6 (0.4–1.0)		0.6 (0.4–1.1)	
≥7	0.7 (0.4–1.1)		0.7 (0.4–1.3)	
Total number of abortions		0.06*		0.007*
0–2 (ref)	1.0		1.0	
3–4	1.5 (1.0–2.1)		1.7 (1.1–2.5)	
≥5	1.4 (0.8–2.4)		1.8 (1.0–3.6)	
Religion		0.04		0.09
Islam (ref)	1.0		1.0	
Christian	1.7 (1.0–2.8)		1.5 (0.9–2.5)	
Education		0.5		
None (ref)	1.0			
Quaranic	0.8 (0.1–6.3)			
Primary	(0.5–2.7)			
Secondary	(0.7–3.4)			
Tertiary	1.3 (0.6–2.8)			
Marital Status		0.08		1.0
Single (ref)	1.0		1.0	
Married	0.5 (0.4–0.8)		(0.6–1.2)	
Widowed	0.5 (0.3–0.8)		(0.6–1.6)	
Divorced	0.7 (0.4–1.3)		(0.6–1.9)	
Cohabiting	0.6 (0.1–4.1)		0.6 (0.1–4.1)	
Occupation		0.6		
Employed (ref)	1.0			
Unemployed	(1.4–4.2)			
Student	(0.4–5.5)			
Other	1.0 (0.5–2.1)			
N/A	1.4 (0.4–5.3)			
Contraceptive use	1.0 (0.7–1.4)	1.0		
Hormonal contraceptives	0.6 (0.1–3.8)	0.5		
Intrauterine devices	1.5 (0.5–4.5)	0.5		
Barrier contraceptives	1.2 (0.8–1.7)	0.4		
**Clinical characteristics**				
BMI (kg/m^2^)		0.8		
<18.5 (ref)	1.0			
18.5–24.9	(0.7–1.5)			
25–29.9	0.6 (0.2–2.5)			
≥30	1.0 (0.5–1.8)			
Vaginal wall abnormality		0.0006		0.0007
None (ref)	1.0		1.0	
Other	2.0 (1.3–2.9)		1.9 (1.3–2.8)	
Last CD4 count (per mm^3^)		<0.0001*		<0.0001*
<300 (ref)	1.0		1.0	
300–<450	0.5 (0.3–0.7)		(0.3–0.7)	
450–<650	0.5 (0.3–0.7)		0.5 (0.3–0.8)	
≥650	0.3 (0.2–0.5)		0.3 (0.2–0.6)	
WHO stage		1.0		
Stage 1 (ref)	1.0			
Stage 2	(0.2–1.4)			
Stage 3	1.3 (0.6–2.7)			
Stage 4	1.6 (0.2–10.2)			
ART Regimen currently on		0.6		
TDF-containing ART	1.4(1.0–2.0)			
ZDV-containing ART	(1.0–2.2)			
d4T-containing ART	0.9 (0.4–2.1)			

*p-value for trend using median value of each category.

## Discussion

In this study of cervical cancer risk factors in HIV-positive Nigerian women, we found a 6% prevalence of cervical pre-cancer and cancer. This finding is inconsistent with previous reports from other Sub-Saharan African countries where a higher prevalence of pre-cancer and cancer has been reported [31-38]. Studies conducted in Rwanda, Kenya, South Africa, Uganda and Zambia reported prevalence of cervical pre-cancer and cancer among HIV-positive women of 24.3%, 26.7%, 66.3%, 73.0% and 76%, respectively [31-35].

In order to address these inconsistencies, knowing that the age-standardized incidence of cervical cancer in Nigeria is 34.5 per 100,000 people/year and this has not changed significantly in the last few decades [3], that 20% of cervical abnormalities if untreated progress to carcinoma, and assuming that the average duration from a cervical abnormality to cancer is about 15 years given that the average duration of cervical cancer from the time of infection with the HPV to cervical cancer has been estimated at 30 years [39], it is possible to estimate the expected prevalence of cervical abnormalities. The relationship between the incidence, I, and the point prevalence of disease, p, can be used to calculate the expected prevalence of cervical abnormalities as follows: $p=\frac{I\bar{D}}{1+I\bar{D}}$, where $\bar{D}$ is the average duration of the disease [40]. Applying this formula and based on the assumptions made above, an expected prevalence of 2.5% is obtained. Further assumptions based on the sensitivity of VIA/VILI will increase this prevalence estimate. It is also easy to see how, with small departures from these assumptions, the prevalence of cervical abnormality observed in the study could have been easily obtained. For example, if only 10% of untreated cervical abnormalities in this setting progress to frank cancer, the expected prevalence of cervical abnormalities would be 4.9%. It is noted that the incidence of cervical cancer in East Africa is about 50% higher than in West Africa [1]. For prevalence to be as high as that reported in some of the other studies in Sub-Saharan African, such as 27% as reported in a recent study among HIV-infected women in Kenya, the cervical cancer incidence rate would have to be about 493/100,000 people/year; approximately 10-fold greater than what has been observed in African population-based registries. Thus, it is probable that the cervical pre-cancer and cancer prevalence obtained in these other studies were over-estimated and the results of the present study are likely to be much closer to the true value.

The higher prevalence reported by some of these other studies may also be partly explained by differences in the sexual practices of the women studied [2]. Having multiple sexual partners increases the risk of acquiring HPV, and in turn, the development of cervical pre-cancer and cancer. In Cote D’Ivoire, 56% of the women had ≥5 lifetime sexual partners and in South Africa the median number of sexual partners was 4, while in the present study 96% of women reported ≤ 2 sexual partners [2,33,41]. It is, however, unlikely that these differences are sufficient to account for such high rates of prevalence as those reported in some of the previous studies. The limitations of self-reported sexual practices data are noted, but it is not believed that this would differ systematically among Sub-Saharan countries [42].

This study is similar to other studies where participants are recruited through HIV and ART clinics [5,32,33,43]. The median age of women in this study was similar to that of women in other studies [32,33,44]. In general, women in the current study had higher CD4 counts than women in other studies, as all HIV-infected women in this study had initiated ART treatment [32,33,44].

The prevalence and incidence of cervical cancer is only marginally affected by HIV/AIDS [2]. Since the introduction of anti-retroviral therapy, the incidence of cervical cancer has not decreased substantially, unlike that of other AIDS-defining cancers such as Kaposi’s sarcoma and non-Hodgkin’s lymphoma. This suggests that immunosuppression caused by HIV infection is only marginally associated, at best, with the progression to cervical cancer [9,14-18]. The lack of a decrease in the incidence of cervical cancer in this era of effective ART is supported by data from HIV/AIDS cohorts in developed countries [18,45,46]. This finding may be partially explained by the lack of impact of immunosuppression on carcinogenetic HPV, a necessary but not sufficient cause of cervical pre-cancer and cancer [44,47].

The majority of the women in this study were aged between 30 and 39 (55%), and the median age observed for an invasive cancer diagnosis or positive screening result was 32 years, which is about 10 years younger than the median age of 47 years for cervical cancer diagnosis in the general population [48]. This result is similar to that of other studies from Africa, which showed HIV-positive women presenting with cervical pre-cancer and cancer at an earlier age. In Kenya and South Africa, HIV-positive women with invasive cervical cancer were on average about 10 years younger compared with women who were HIV-negative [49,50]. One hypothesis is that unsafe sexual behavior puts women at risk of both HPV and HIV, and that this may explain the younger age of presentation of HIV-infected women [49]. However, given the results from HIV cohorts in developed countries that suggest a limited role of HIV-associated immunosuppression in cervical carcinogenesis [18,45,46], the differences in age between HIV-positive and HIV-negative people may be a reflection of the lower life expectancy of persons living with HIV/AIDS in Africa.

The results of this study suggest that HIV-infected women with higher CD4 cell counts were at a reduced risk for invasive cancer diagnosis or a positive VIA result. This result agrees with findings from other studies. For example, in Rwanda, an inverse association was found between the CD4 count and cervical pre-cancer among HIV-infected women [44]. Harris et al. found that HIV-negative and HIV-positive women with a CD4 cell count of greater than 500 cells per mm^3^ had low incidence of cervical pre-cancer compared with those with lower CD4 counts [51]. These findings may reflect competing risks in HIV-positive patients with low CD4 counts, and the need to focus more on immune reconstitution in that population. It also appears that once pre-cancerous lesions develop, disease progression is not affected by the CD4 count, consistent with the marginal role previously reported for immunosuppression in disease progression [52].

We also found the risk for cervical pre-cancer and cancer to be associated with age (≥40 years) which supports the natural history of cervical carcinogenesis from hrHPV infection through cervical dysplasia, pre-cancer and cancer; having five or more abortions and the presence of vaginal wall abnormalities suggesting a generalized increased risk of sexually transmitted diseases in women who have cervical pre-cancer and cancer. The non-linearity in the relationship between age and screening status may be due to the natural history of cervical carcinogenesis where infection needs to persist before engendering malignant change [53].

As this was a cross-sectional study it was not possible to explore cause-and-effect relationships. We did not perform HPV DNA testing, cytology or biopsies to confirm the result of visual inspections; as a result, visually inapparent pre-cancerous lesions could have been missed. Also, since this study was conducted among HIV-infected women on ART, the results may not be generalizable to uninfected women. Nevertheless, we believe this study demonstrates the risk factors of positive cervical cancer screening test in this population.

## Conclusion

In conclusion, HIV-positive Nigerian women are at a marginally increased risk for cervical pre-cancer and cancer. Our study highlight the importance of screening HIV-positive women in Nigeria for cervical cancer. The screen and treat approach using VIA/VILI has been shown to be effective for the detection of cervical pre-cancer and cancer, and incorporation of immediate treatment where applicable is associated with reduced loss to follow-up. This can also be rapidly scaled up in resource-constrained settings and be incorporated into HIV treatment programs. Our results demonstrate the need for more epidemiological studies of cervical pre-cancer and cancer among HIV-positive, indeed all women in Africa and an improved understanding of incidence and risk factors.

### Consent

Written informed consent was obtained from the patient for publication of this report and any accompanying images.

## Competing interests

The authors declare that they have no competing interests.

## Authors' contributions

UO conducted the data analysis and drafted the manuscript. MA implemented the study, collected data, assisted with data analysis and drafting of the manuscript. ZM implemented the study, collected data, assisted with data analysis and drafting of the manuscript. IL worked on study implementation and data collection. RO supervised clinical data collection and assisted with drafting the manuscript. OO supervised clinical data collection and assisted with drafting the manuscript. PD supervised clinical data collection and assisted with drafting the manuscript. DS supervised data analysis and writing of the manuscript. WB supervised clinical data collection and assisted with drafting the manuscript. CA conceived the study, participated in study implementation, data collection, data analysis and drafting of the manuscript. All authors read and approved the final manuscript.

## Pre-publication history

The pre-publication history for this paper can be accessed here:

http://www.biomedcentral.com/1471-2458/13/582/prepub
